# An intelligent framework for dynamic modeling of therapeutic response using clinical compliance data

**DOI:** 10.3389/fphar.2025.1631599

**Published:** 2025-12-01

**Authors:** Xinyi Wang, Haoran Xie

**Affiliations:** 1 Columbia University, New York, NY, United States; 2 Information Science and Technology College, Dalian Maritime University, Dalian, China

**Keywords:** dynamic medication modeling, probabilistic inference, medication adherence, Bayesian transformer, clinical decision support

## Abstract

**Introduction:**

The increasing availability of real-world clinical compliance data provides unprecedented opportunities to model medication behaviors dynamically and personalize treatment strategies. However, the complex, heterogeneous, and often incomplete nature of these data presents significant modeling challenges, particularly for capturing medication nonadherence, patient-specific therapeutic dynamics, and drug interaction effects. Existing approaches, including statistical regression models and rule-based decision systems, often fail to capture the high-dimensional, temporally-evolving, and probabilistic characteristics inherent in medication trajectories, limiting their effectiveness in precision medicine and policy simulation contexts.

**Methods:**

To address these limitations, we propose a novel intelligent computing framework that unifies probabilistic graphical modeling, deep temporal inference, and domain-informed strategy design. Our approach is instantiated in the Hierarchical Therapeutic Transformer (HTT), a Bayesian transformer-based model that captures therapeutic state transitions via structured latent variables and medication-aware attention mechanisms. Furthermore, we introduce the Pharmacovigilant Inductive Strategy (PIS), a training paradigm that integrates pharmacological priors, adaptive quantification, and entropy-driven curriculum learning to enhance robustness and generalizability. Our method effectively models dose-response variability, accounts for clinical data missingness, and generalizes across cohorts through a hierarchical latent prior framework.

**Results and discussion:**

Experimental evaluations demonstrate that our system achieves state-of-the-art performance in predicting adherence patterns and clinical outcomes across diverse datasets, aligning with current advances in medication adherence modeling and probabilistic health informatics. This work provides a rigorous, interpretable, and scalable foundation for real-time decision support in pharmacotherapy, contributing to the broader goals of personalized medicine, drug safety monitoring, and computational clinical reasoning.

## 1 Introduction

The increasing complexity of clinical treatments and the individual variability in drug responses demand innovative computational approaches that go beyond static modeling. Drug behavior is influenced not only by physiological and molecular factors but also by dynamic clinical compliance data, such as dosage timing, adherence patterns, and patient-specific behaviors. Modeling such complexity has drawn inspiration from sequence learning architectures originally developed in vision applications, such as transformers, due to their capacity for capturing evolving data patterns (Maurício et al., 2023). Not only does the integration of compliance data into drug modeling allow for more precise prediction of pharmacokinetics and pharmacodynamics, but it also enhances the potential for personalized treatment planning and outcome prediction (Vrijens et al., 2012). With the rise of real-world data collected from electronic health records and wearable devices, incorporating clinical compliance into intelligent modeling frameworks enables real-time, adaptive simulations that better reflect real-life scenarios (Brown and Bussell, 2011). Therefore, developing an intelligent computing framework that leverages clinical compliance data is essential for capturing the dynamic nature of drug behavior and ultimately improving the efficacy and safety of pharmacotherapy (Wang et al., 2022). Recent studies have shown that nonadherence to prescribed medications remains one of the most under-addressed challenges in pharmacotherapy. It contributes significantly to treatment failure and worsens health outcomes. Moreover, poor adherence increases healthcare costs due to avoidable complications and hospitalizations. Understanding and modeling adherence dynamics is therefore essential to predict real-world drug exposure. Such models can help bridge the gap between prescription intent and actual patient behavior (Osterberg and Blaschke, 2005). Early modeling strategies focused on encoding well-established pharmacological relationships using deterministic frameworks grounded in curated knowledge and simplified assumptions (Tian et al., 2020). These models offered clear interpretability and helped simulate basic absorption, distribution, metabolism, and excretion (ADME) processes under controlled conditions (Yang et al., 2021). While these representations contributed valuable insights into drug mechanisms, they were often too rigid to capture patient-specific variability or adjust to real-time changes in adherence behavior (Hong et al., 2020). Their inability to accommodate evolving or noisy clinical data limited their utility in dynamic, personalized treatment settings (Sun et al., 2022). These challenges catalyzed a methodological shift toward more flexible paradigms that could incorporate richer behavioral and temporal patterns.

Building upon these foundations, newer computational models began to explore more adaptive strategies capable of learning from observed clinical phenomena (Rao et al., 2021). These approaches combined empirical data with structural modeling, incorporating statistical correlations and input features derived from large clinical datasets (Mai et al., 2021). Such frameworks allowed the integration of compliance indicators–like dose irregularities or temporal gaps in treatment–into outcome prediction pipelines, enabling more refined estimations of drug efficacy (Azizi et al., 2021). However, these methods often relied on domain-specific tuning and struggled with capturing long-range dependencies or sequential effects across time windows (Li et al., 2020). As the demand for continuous, patient-centered modeling increased, attention turned to temporal algorithms capable of handling evolving data contexts. Architectures such as ResMLP, though originally designed for image classification, have influenced the design of efficient feedforward mechanisms in time-series prediction models (Touvron et al., 2021).

Recent advances have led to the convergence of sequence modeling architectures and pretraining strategies for biomedical applications (Bhojanapalli et al., 2021). By leveraging time-aware neural networks and pretrained representations–many of which were initially established in medical imaging contexts–researchers have adapted such methods to dynamically adjust predictions based on incoming compliance data streams (Kim et al., 2022). These models excel at tracking longitudinal variations and recognizing subtle adherence patterns that may influence pharmacological trajectories. Nevertheless, despite their adaptive power, they often lack the transparency required for clinical interpretation and may fail to incorporate mechanistic knowledge critical for understanding drug interactions. As such, there is growing recognition that future solutions should merge structured pharmacological understanding with real-time data processing, paving the way for hybrid frameworks that combine interpretability with adaptability in clinical pharmacotherapy.

Based on the limitations of symbolic rigidity, shallow learning’s task-specificity, and deep learning’s opacity, we propose an intelligent computing framework that synergistically integrates clinical compliance data with dynamic modeling of drug behavior. Our method unifies temporal representation learning with domain-aware pharmacological reasoning, allowing for continuous, interpretable, and context-sensitive drug simulations. By leveraging both real-time adherence signals and pretrained medical embeddings, this approach captures patient-specific deviations and drug interactions over time, offering a robust and adaptive alternative to static or purely data-driven models. Furthermore, the incorporation of modular components enhances the framework’s scalability and adaptability to various therapeutic areas and patient cohorts. This not only bridges the gap between generalizability and personalization but also allows for real-world applications such as dosage optimization, risk prediction, and treatment adherence monitoring. Ultimately, this framework paves the way for more effective, safe, and patient-centered pharmacological interventions.

Our approach draws methodological inspiration from architectural innovations in visual computing, including transformers, self-supervised learning, and efficient feedforward networks, which we adapt to the context of temporal clinical modeling and compliance-aware decision support. The proposed approach offers several significant benefits:

• We introduce a novel modular architecture that unites real-time compliance tracking with pretrained pharmacological knowledge representations, enabling continuous updates to individualized therapeutic response simulations.
• Our method supports multi-scenario deployment, offering high efficiency and generalizability across diverse drug types, populations, and adherence patterns.
• Experimental results on real-world clinical datasets demonstrate superior predictive accuracy, dynamic adaptability, and interpretability compared to baseline symbolic and deep models.

## 2 Related work

### 2.1 Clinical compliance data modeling

Modeling clinical compliance data requires nuanced understanding of both structured and unstructured medical records, adherence patterns, and behavioral variability among patients (Luo et al., 2025). A significant body of research focuses on electronic health records (EHRs) and their use in identifying non-adherence signals, medication intake trends, and longitudinal tracking of clinical outcomes (Simpson et al., 2006). Traditional approaches have relied on statistical modeling, such as logistic regression and Cox proportional hazards models, to analyze medication adherence and its correlation with therapeutic success or failure. However, these models often fail to capture the temporal and contextual intricacies inherent in real-world compliance behaviors (Zhang et al., 2020). Recent advancements integrate temporal modeling techniques using deep learning, particularly recurrent neural networks (RNNs), gated recurrent units (GRUs), and transformers, to learn compliance sequences from timestamped medication logs. These methods allow for personalized adherence and real-time detection of deviation patterns (Kardas et al., 2013). Furthermore, research has explored multimodal data fusion, incorporating clinical notes, pharmacy refill records, wearable sensor data, and patient-reported outcomes to enrich compliance modeling. Natural language processing (NLP) is instrumental in parsing physician notes and discharge summaries to extract adherence-related information (Zhu et al., 2020). Moreover, federated learning and privacy-preserving computation paradigms have emerged to enable large-scale training of compliance models without compromising patient privacy (Brisimi et al., 2018). These distributed learning frameworks are increasingly critical in clinical settings where data sensitivity and regulatory compliance are paramount Ashtiani et al. (2021). Collectively, the integration of deep temporal models, multimodal data fusion, and privacy-aware computing underpins the current direction of intelligent systems for modeling clinical compliance data (Masana et al., 2020).

### 2.2 Drug behavior dynamics modeling

Understanding the dynamic behavior of drugs within the human body involves capturing complex pharmacokinetic (PK) and pharmacodynamic (PD) interactions (Zhang et al., 2025). Conventional models, such as compartmental models and physiologically based pharmacokinetic (PBPK) models, have long served as the foundation for quantifying drug absorption, distribution, metabolism, and excretion (ADME). While these models offer interpretability, they often require extensive domain-specific parameter tuning and may struggle to generalize across patient populations or new drug compounds (Jiao et al., 2010). In recent years, machine learning has transformed drug behavior modeling by enabling data-driven inference of PK/PD relationships (Sheykhmousa et al., 2020). Neural differential equations and hybrid modeling frameworks combine mechanistic insights with the flexibility of deep learning to accommodate inter-individual variability and complex dose-response relationships. These approaches dynamically adjust to real-time patient data, improving predictive accuracy and personalization (Mascarenhas and l Agarwal, 2021). Studies have also introduced graph neural networks (GNNs) and attention mechanisms to model molecular-level drug interactions and their downstream effects (Zhang et al., 2022). These architectures capture structural and relational information, facilitating the understanding of drug-drug interactions and polypharmacy effects (Dai and Gao, 2021). Coupling such models with clinical compliance data introduces a feedback mechanism that reflects the true drug exposure experienced by patients, rather than relying solely on prescribed regimens (Taori et al., 2020). This integrative modeling direction supports adaptive dosing strategies and real-time therapeutic monitoring. It lays the groundwork for intelligent systems that not only predict drug behavior but also suggest compliance-aware treatment optimizations based on patient-specific dynamics.

### 2.3 AI frameworks in clinical intelligence

Artificial intelligence frameworks designed for clinical intelligence aim to support decision-making, treatment personalization, and predictive modeling (Williams et al., 2009). These systems must handle heterogeneous data types, comply with regulatory standards, and deliver interpretable outputs to support clinical trust and adoption. Contemporary frameworks employ modular architectures that integrate data ingestion, preprocessing, feature engineering, and model interpretation pipelines (Brown et al., 2008). Graph-based and knowledge-infused architectures have become prevalent, leveraging medical ontologies such as SNOMED CT and UMLS to enhance data contextualization (Peng et al., 2022). In compliance-aware drug modeling, these frameworks facilitate the mapping of medication events to standardized terminologies, enabling more accurate cross-patient analysis and data harmonization (Bazi et al., 2021). Reinforcement learning (RL) has also seen application in adaptive treatment planning, where reward structures incorporate compliance fidelity and drug efficacy (Zheng et al., 2022). Explainability techniques, such as SHAP (SHapley Additive exPlanations) and attention visualizations, are critical for unveiling model behavior, especially in high-stakes domains like drug response modeling. Model accountability is further enforced through audit trails, provenance tracking, and validation against clinical benchmarks (Dong et al., 2022). Several platforms have integrated real-time compliance tracking into their AI workflows. These include mobile health (mHealth) solutions, edge computing for wearable integration, and cloud-based AI inference services. As these frameworks mature, they increasingly adopt ethical AI principles, including bias mitigation, fairness auditing, and robust handling of missing or noisy data. These developments contribute to a more holistic, intelligent framework capable of dynamically modeling drug behavior grounded in real-world clinical compliance.

## 3 Methods

### 3.1 Overview

Modeling medication-related phenomena poses a unique set of challenges within the machine learning community, particularly in the context of probabilistic inference and domain-specific representation. Unlike general image or text datasets, medication data encapsulate a wide array of intricate, structured, and often incomplete information, ranging from patient-level variability to pharmacological interactions and temporal dynamics. This subsection provides an overview of our proposed methodology for addressing these complexities.

Our starting point is a probabilistic framework for modeling patient-specific therapeutic processes over time. Unlike classical classification tasks, medication modeling requires handling uncertainty at both the observation and latent levels, including drug effects, side effects, patient adherence, and dosage variability. We formulate this as a hierarchical latent variable model, where unobserved states evolve over time and influence observable clinical outcomes. The model structure follows that of probabilistic temporal inference frameworks Rezende et al. (2014); Vaswani et al. (2017), which have been widely used in dynamic state-space modeling. We incorporate domain-specific priors by conditioning latent transitions on medication encodings, allowing the model to simulate personalized responses and adherence-aware treatment trajectories. This design supports interpretable, uncertainty-aware modeling in complex real-world clinical environments. Section 3.2 sets the stage by formalizing this task within a probabilistic graphical model framework.

We define a multi-level latent state space in which medication effects, patient responses, and clinical observations are represented as interdependent random variables, following the structure of probabilistic graphical models commonly used in temporal inference frameworks (Koller and Friedman, 2009).

This design is conceptually inspired by hierarchical graphical models, such as those proposed by Friston et al., which have been widely applied to dynamic inference in clinical and physiological settings (Friston et al., 2018). These models provide a principled framework for representing temporally-evolving hidden states and their interactions with observable variables in noisy, real-world environments. Building on this foundation, we extend the graphical paradigm to account for domain-specific structures in pharmacological contexts, particularly medication adherence and drug interaction effects. Our adaptation introduces adherence-aware latent transitions and personalized therapeutic priors to improve fidelity in modeling real-world drug behavior. In contrast to generic temporal models, our framework explicitly captures the influence of medication-specific dynamics on patient outcomes, offering both statistical expressiveness and clinical interpretability. This symbolic foundation not only allows us to capture uncertainty but also enables a principled approach to model interpretability and downstream clinical decision-making. The complexity of medication dynamics necessitates an expressive yet tractable modeling apparatus. Section 3.3 introduces our architecture, which we term the Hierarchical Therapeutic Transformer (HTT). This architecture is built upon a foundation of Bayesian neural computation and incorporates temporal attention mechanisms, enabling it to flexibly model long-range dependencies between drug administration events and patient outcomes. Unlike standard Bayesian neural networks, HTT integrates structured pharmacological priors and heterogeneous clinical contexts through a variational posterior defined over a sequence of latent therapeutic states. Furthermore, to mitigate the storage inefficiencies of MCMC sampling, we incorporate a generative compression module inspired by adversarial distillation paradigms. This not only reduces the memory footprint but also enables rapid posterior sampling at inference time without loss of uncertainty fidelity. Recognizing that real-world medication modeling transcends mere model design, Section 3.4 introduces our Pharmacovigilant Inductive Strategy (PIS). The strategy orchestrates the interplay between domain-specific priors, observational biases, and uncertainty calibration in a coherent training paradigm. In particular, we address the challenges of distributional shift due to cohort heterogeneity, missingness in longitudinal electronic health records, and the nuanced semantics of clinical endpoints. Our strategy employs an adaptive objective that balances epistemic and aleatoric uncertainty across subpopulations, leveraging both Bayesian ensemble estimates and entropy-based active sampling. Importantly, PIS is agnostic to specific pharmacological classes, allowing it to generalize across different therapeutic domains with minimal reconfiguration.

### 3.2 Preliminaries

Terminology Clarification: To ensure clarity and consistency across Sections 3.2–3.4, we summarize key variables, notations, and technical components used in our modeling framework in Table 1. This supports interpretability and improves accessibility to readers unfamiliar with latent state modeling or curriculum-based training. Medication modeling centers on representing and reasoning about the probabilistic effects of pharmaceutical interventions across diverse patient populations. The aim is to develop a structured inferential framework that captures uncertainty, individual heterogeneity, and the dynamic evolution of patient states under varying medication regimes. In this section, we present a formal mathematical characterization of this problem, establishing the probabilistic foundations and notational conventions that will underlie the remainder of our proposed methodology.

**TABLE 1:** Glossary of key terms and notations.

Symbol/Term	Definition
X	Observable patient data (vital signs, lab tests, notes, etc.)
A	Medication actions (dosage, frequency, route of administration)
Z	Latent therapeutic states inferred from observed data
$c^{\left(i\right)}$	Patient-level covariates (age, sex, comorbidities)
$\epsilon_{t}$	Residual stochastic noise capturing transient variations
g	Global latent variable encoding static patient traits
$H\left[z_{t}\right]$	Entropy of the latent state $z_{t}$
$\omega_{t}$	Inverse-variance weight for uncertainty-aware optimization
$w_{t}^{\mathrm{ent}}$	Curriculum sampling weight derived from $H\left[z_{t}\right]$
$\lambda_{\tau }$	Task-specific loss weight
Φ	Embedding matrix for pharmacological entities
$L_{\mathrm{graph}}$	Graph-based regularization term enforcing drug similarity
Curriculum Sampling	Training strategy that starts with low-uncertainty samples
Domain Alignment	Modeling adjustment based on population domain d
Reparameterization Trick	Method to enable gradients through stochastic variables
Medication-Informed Generation	Attention-driven modulation of $z_{t}$ by drug embeddings

Let X denote the space of observable patient data, including vital signs, laboratory results, and clinical notes. Let A denote the space of discrete or continuous medication actions, such as dosage amount (10 mg), administration frequency (twice daily), and delivery method (oral or intravenous). These actions represent externally administered interventions intended to modify the patient’s therapeutic state. For a given patient i , we observe a time-indexed sequence of states and actions (Equation 1):

$$D^{\left(i\right)}={\left\{\left(x_{t}^{\left(i\right)},a_{t}^{\left(i\right)}\right)\right\}}_{t=1}^{T^{\left(i\right)}}$$ (1)

here, $D^{\left(i\right)}$ denotes the full observed treatment trajectory for patient i , consisting of sequential clinical observations and corresponding medication actions over time. Where $x_{t}^{\left(i\right)}\in X$ represents the observed clinical state at time t , and $a_{t}^{\left(i\right)}\in A$ denotes the administered medication. The length $T^{\left(i\right)}$ varies per patient.

We introduce a latent variable $z_{t}^{\left(i\right)}\in Z$ to denote the unobserved therapeutic state, reflecting a patient’s internal physiological response to medication. The joint generative model over a patient trajectory is formulated as Equation 2:

$$p\left({\left\{x_{t},z_{t},a_{t}\right\}}_{t=1}^{T}\right)=p\left(z_{1}\right)\overset{T}{\underset{t=1}{\prod }}p\left(x_{t}|z_{t}\right)p\left(a_{t}|z_{t}\right)p\left(z_{t+1}|z_{t},a_{t}\right)$$ (2)

where $p\left(z_{1}\right)$ denotes the prior distribution over initial therapeutic states, $p\left(x_{t}|z_{t}\right)$ captures the observation model, $p\left(a_{t}|z_{t}\right)$ reflects the medication policy, and $p\left(z_{t+1}|z_{t},a_{t}\right)$ governs state transitions under medication.

To model the influence of multiple concurrent medications, we define Equation 3:

$$a_{t}=\left\{a_{t}^{\left(1\right)},a_{t}^{\left(2\right)},\ldots ,a_{t}^{\left(M\right)}\right\},\;a_{t}^{\left(m\right)}\in R_{\geq 0}$$ (3)

where M is the number of active ingredients or drugs. We assume an additive interaction model in the latent space (Equation 4):

$$z_{t+1}=f\left(z_{t}\right)+\overset{M}{\underset{m=1}{\sum }}g_{m}\left(a_{t}^{\left(m\right)},z_{t}\right)+\epsilon_{t}$$ (4)

with $\epsilon_{t}∼N\left(0,\Sigma_{z}\right)$ representing transition noise, f(⋅) encoding the natural disease progression, and $g_{m}\left(\cdot ,\cdot \right)$ denoting the pharmacodynamic effect of drug m .

Patient heterogeneity is encoded via a static covariate vector $c^{\left(i\right)}\in R^{d}$ capturing demographics, comorbidities, and genotypic traits. The prior over initial therapeutic states is conditioned as (Equation 5):

$$p\left(z_{1}^{\left(i\right)}|c^{\left(i\right)}\right)=N\left(\mu \left(c^{\left(i\right)}\right),\Sigma \left(c^{\left(i\right)}\right)\right)$$ (5)

To account for partial observability and missingness in $\left\{x_{t}\right\}$ , we define a binary mask $m_{t}\in {\left\{0,1\right\}}^{\left|X\right|}$ indicating which features are present. The emission model then becomes (Equation 6):

$$p\left(x_{t}|z_{t},m_{t}\right)=\overset{\left|X\right|}{\underset{j=1}{\prod }}{\left[p\left(x_{t,j}|z_{t}\right)\right]}^{m_{t,j}}$$ (6)

We are interested in modeling the posterior distribution over latent therapeutic states conditioned on observed data (Equation 7):

$$p\left({\left\{z_{t}\right\}}_{t=1}^{T}|{\left\{x_{t},a_{t}\right\}}_{t=1}^{T}\right)$$ (7)

Given the intractability of exact inference in this temporal latent-variable model, our approach approximates the posterior using a structured variational distribution, inspired by sequence-aware variational inference frameworks such as those proposed in deep temporal models (Equation 8) (Fraccaro et al., 2016).

$$q\left({\left\{z_{t}\right\}}_{t=1}^{T}\right)=q\left(z_{1}\right)\overset{T-1}{\underset{t=1}{\prod }}q\left(z_{t+1}|z_{t},x_{t},a_{t}\right)$$ (8)

Temporal dependencies are further enriched through the introduction of retrospective attention. Define an attention window of size w , and a context embedding (Equation 9):

$$h_{t}=\overset{t-1}{\underset{k=t-w}{\sum }}\alpha_{t,k}\cdot \phi \left(x_{k}\right),\;\alpha_{t,k}=\frac{\exp\left(e_{t,k}\right)}{\sum_{j}\exp\left(e_{t,j}\right)},\;e_{t,k}=\psi \left(x_{t},x_{k}\right)$$ (9)

where ϕ(⋅) and ψ(⋅,⋅) are learnable encoders and compatibility functions, respectively.

In Figure 2, the variable H refers to the same context-enhanced embedding $h_{t}$ defined in Equation 9. Both denote the output of the retrospective attention mechanism that summarizes temporal clinical signals. Specifically, $h_{t}$ aggregates previous observations using attention weights derived from pairwise similarity functions $\psi \left(x_{t},x_{k}\right)$ across a sliding window. This allows the model to assign adaptive importance to each prior time step. Although Figure 2 uses a simplified visual notation for clarity, the semantics are consistent with the formal temporal embedding defined in our inference framework. This clarification ensures that the architectural diagram and the probabilistic formulation remain fully aligned.

### 3.3 Hierarchical therapeutic transformer

**FIGURE 1:**
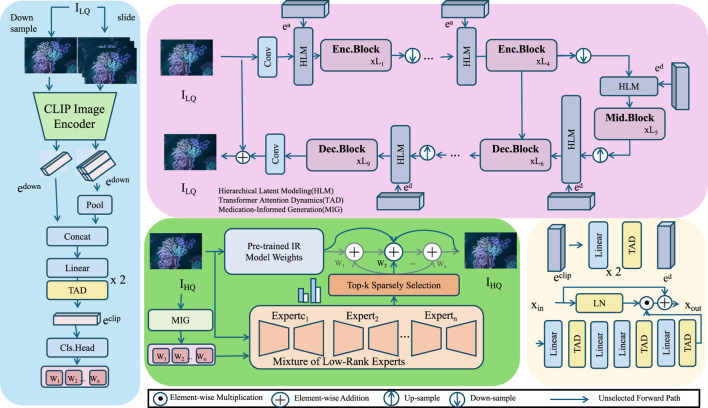
Hierarchical Therapeutic Transformer (HTT) architecture diagram. Overview of the Hierarchical Therapeutic Transformer (HTT) architecture, including multimodal encoders, hierarchical latent modeling, and medication-specific inference modules. See Section 3.3 for detailed description.

The Hierarchical therapeutic transformer (HTT) is a novel latent sequence model that builds upon two foundational concepts: hierarchical latent variable modeling and attention-based temporal architectures. Hierarchical inference structures enable the representation of multiscale latent states corresponding to global patient traits, therapeutic dynamics, and local perturbations (Friston, 2008). In parallel, attention mechanisms from the Transformer architecture offer a flexible means to model non-Markovian dependencies in medical sequences (Vaswani et al., 2017). Our proposed HTT integrates these ideas by organizing latent states into a three-tier structure ( g , $z_{t}$ , $\epsilon_{t}$ ), each modulated by medication-conditioned attention. This allows the model to represent personalized therapeutic effects while maintaining uncertainty awareness and temporal expressiveness. The core modeling innovation of this work is the Hierarchical Therapeutic Transformer (HTT), a deep generative model tailored to represent medication effects through a structured probabilistic temporal process. HTT is designed to simultaneously encode patient-specific therapeutic trajectories and medication-response dependencies using hierarchical latent variables, attention-driven temporal abstraction, and generative compression mechanisms. This section details the architectural components of HTT, its probabilistic semantics, and the interleaving of pharmacological priors within its stochastic generative process. Figure 1 illustrates the full architecture of the Hierarchical Therapeutic Transformer (HTT). The left portion of the figure includes a CLIP-based image encoder for multimodal physiological feature extraction, which serves as a supplemental input when available (medical imaging). This is followed by a hierarchical latent modeling structure (HLM), implemented via encoder-decoder Transformer blocks that capture multiscale therapeutic state transitions. Temporal abstraction is achieved through the Transformer Attention Dynamics (TAD) module, which models delayed and long-range medication effects. On the output side, a Mixture-of-Experts (MoE) framework enables medication-specific inference pathways, enhancing interpretability and efficiency. These components are jointly optimized under a variational Bayesian framework, with task-specific decoders instantiated for downstream prediction tasks.

#### 3.3.1 Hierarchical latent modeling

The foundation of the Hierarchical Therapeutic Transformer (HTT) lies in its ability to represent heterogeneous clinical data through a deeply structured probabilistic process that is both temporally coherent and semantically aligned with medical reasoning. Central to this architecture is the introduction of a three-tiered hierarchy of latent variables that capture multi-scale variations in patient response to pharmacological interventions. The highest level of abstraction, encoded by a global latent variable $g\in R^{d_{g}}$ , encapsulates enduring patient-specific characteristics that modulate the trajectory of treatment over time. This includes intrinsic factors such as genetic predisposition, comorbidities, and long-term physiological markers, all of which influence therapeutic response but remain temporally invariant across the sequence. At the intermediate level, the latent trajectory ${\left\{z_{t}\right\}}_{t=1}^{T}$ governs the temporal evolution of the patient’s therapeutic state, reflecting how treatment effects unfold dynamically in response to administered medications and underlying conditions. These latent states are not directly observable but are inferred from noisy clinical measurements, and they serve as the key representational core for decision-making and simulation. To capture short-term fluctuations and data-level stochasticity, a local residual process ${\left\{\epsilon_{t}\right\}}_{t=1}^{T}$ is introduced, modeling transient noise and idiosyncratic variations unaccounted for by the slower therapeutic dynamics. The hierarchical joint distribution over clinical observations $x_{1:T}$ and latent variables conditioned on the medication sequence $a_{1:T}$ can thus be formulated as follows (Equation 10):

$$p\left(x_{1:T},z_{1:T},\epsilon_{1:T},g|a_{1:T}\right)=p\left(g\right)\overset{T}{\underset{t=1}{\prod }}p\left(z_{t}|z_{t-1},a_{t-1},g\right)p\left(\epsilon_{t}\right)p\left(x_{t}|z_{t},\epsilon_{t}\right)$$ (10)

The initialization follows standard Gaussian priors, where $z_{0}∼N\left(0,I\right)$ and p(g)=N(0,I) , reflecting uninformative assumptions about the system before any data is observed. Transition dynamics within the latent therapeutic state space are governed by a non-linear stochastic process, parameterized via a recurrent architecture that integrates past therapeutic state, prior medication action, and global patient profile. Each latent state $z_{t}$ is modeled as a Gaussian random variable whose mean and variance depend on this historical context, and it is sampled using the reparameterization trick Kingma and Welling (2014), which enables gradient-based optimization by expressing stochastic sampling as a differentiable transformation of noise. Here, the noise $\epsilon_{t}$ is sampled from a standard normal distribution N(0,I) , introducing controlled stochasticity into the latent variable. This design separates randomness from the learned parameters $\mu_{t}$ and $\sigma_{t}$ , enabling differentiable sampling. As a result, gradients can flow through the stochastic layer during backpropagation. This mechanism is critical for optimizing models with latent variables in an end-to-end fashion. It ensures that the model can explore variability while remaining compatible with gradient-based learning. Such a property is fundamental in modern variational inference frameworks (Equation 11).

$$z_{t}=\mu_{t}+\sigma_{t}⊙\xi_{t},\;\xi_{t}∼N\left(0,I\right),\;\left[\mu_{t},\log\sigma_{t}\right]={\text{GRU}}_{\theta }\left(\left[z_{t-1},a_{t-1},\phi_{g}\left(g\right)\right]\right)$$ (11)

We assume that clinical observations follow an isotropic Gaussian likelihood distribution $p\left(x_{t}|z_{t},\epsilon_{t}\right)=N\left(x_{t};f_{\mu }\left(z_{t},\epsilon_{t}\right),\Sigma_{x}\right)$ , which simplifies the generative model while capturing measurement noise. This assumption is common in deep latent-variable models for high-dimensional data Doersch (2016), allowing the decoder to express uncertainty in clinical measurements while remaining computationally tractable. Although the isotropic assumption neglects inter-feature correlations, it is a widely accepted simplification in deep probabilistic models due to its computational efficiency. In many biomedical settings, especially when data is noisy or limited, assuming conditional independence and equal variance across dimensions allows for tractable training without significantly impacting performance. Moreover, when paired with expressive latent representations, the decoder can still capture complex dependencies in the data distribution. This modeling choice is also supported by standard practices in probabilistic machine learning (Equation 12) (Bishop, 2006).

$$p\left(x_{t}|z_{t},\epsilon_{t}\right)=N\left(x_{t};f_{\mu }\left(z_{t},\epsilon_{t}\right),\Sigma_{x}\right)$$ (12)

Inference of the global latent context g is performed via amortized variational inference, using a bi-directional encoder that compresses the full input trajectory $\left\{x_{1:T},a_{1:T}\right\}$ into a Gaussian posterior. This encoder leverages both convolutional and attention-based layers to efficiently integrate temporal and feature-wise correlations across the sequence, yielding variational parameters $\mu_{g}$ and $\Sigma_{g}$ for the posterior approximation (Equation 13):

$$q\left(g|x_{1:T},a_{1:T}\right)=N\left(\mu_{g},\Sigma_{g}\right),\;\left[\mu_{g},\log\Sigma_{g}\right]=f_{\text{enc}}\left(x_{1:T},a_{1:T}\right)$$ (13)

#### 3.3.2 Transformer attention dynamics

To capture the non-Markovian, temporally extended dependencies characteristic of clinical treatment trajectories, the Hierarchical Therapeutic Transformer integrates a self-attention mechanism directly over the latent therapeutic states. This architectural design enables the model to flexibly aggregate information from the full or partial sequence history, thus enhancing its capacity to infer long-term pharmacodynamic effects and patient-specific progression trends. The attention mechanism operates by constructing a context-aware representation of each latent state $z_{t}$ based on its similarity to all preceding latent embeddings $z_{1:t}$ . This similarity is measured via scaled dot-product attention, allowing the model to emphasize or suppress historical influence adaptively. Formally, the attention function computes a weighted sum of value vectors V , scaled by the dot product of queries Q and keys K , normalized through a softmax layer to ensure probabilistic interpretation. The formulation is given by Equation 14:

$$\text{Attn}\left(Q,K,V\right)=\text{softmax}\left(\frac{QK^{⊤}}{\sqrt{d}}\right)V$$ (14)

For each time step t , the self-attention is applied to the latent therapeutic representation $z_{t}$ by projecting it into the query space, and computing attention over the set of all prior latent states. The attention-enhanced representation $Z_{t}$ is then normalized and combined with the original $z_{t}$ via residual connection to preserve information fidelity and ensure stable gradient flow (Equation 15):

$$Z_{t}=\text{LayerNorm}\left(z_{t}+\text{Attn}\left(W_{Q}z_{t},W_{K}z_{1:t},W_{V}z_{1:t}\right)\right)$$ (15)

Where $W_{Q}$ , $W_{K}$ , and $W_{V}$ are learnable matrices mapping inputs into query, key, and value spaces respectively. This form of temporal abstraction enables the model to capture delayed effects and interdependencies among medications and physiological signals over arbitrary horizons, without the inductive bias of fixed window size or sequential recursion. Importantly, this attention mechanism is embedded within a variational inference framework, ensuring that both model complexity and uncertainty are controlled through Bayesian regularization. The full posterior over the latent space is decomposed hierarchically, with joint factorization across global, trajectory-level, and residual variables. For tractability, we define the variational approximation over the full trajectory as Equation 16:

$$q\left(z_{1:T},\epsilon_{1:T},g|x_{1:T},a_{1:T}\right)=q\left(g\right)\overset{T}{\underset{t=1}{\prod }}q\left(z_{t}|z_{<t},x_{\leq t},a_{<t}\right)q\left(\epsilon_{t}|x_{t},z_{t}\right)$$ (16)

The transition encoder for $z_{t}$ is implemented as a gated recurrent unit (GRU), which conditions on the recent context including previous latent state, observation, and administered action. This GRU produces the parameters of a time-specific Gaussian distribution from which $z_{t}$ is sampled via reparameterization (Equation 17):

$$\left[{\tilde{\mu }}_{t},\log{\tilde{\sigma }}_{t}\right]={\text{GRU}}_{\phi }\left(\left[x_{t-1},z_{t-1},a_{t-1}\right]\right)$$ (17)

This recurrent formulation allows for temporal adaptability in the posterior, ensuring that latent transitions remain both expressive and computationally efficient. The interaction between attention-based encoding and variational inference permits the model to reason about future states while retaining calibrated epistemic uncertainty, crucial for clinical interpretability and safety-critical deployment.

#### 3.3.3 Medication-informed generation

**FIGURE 2:**
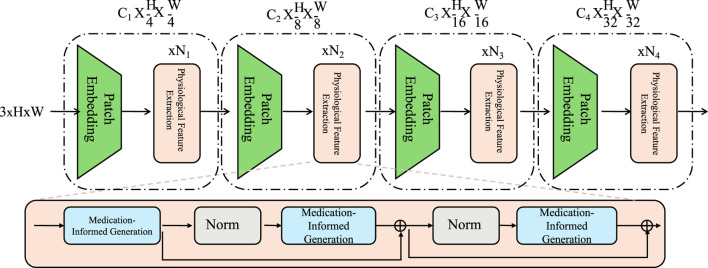
Medication-Informed Generation schematic diagram. Medication-Informed Generation (MIG) module, which integrates drug embeddings into latent state transitions through attention mechanisms. See Section 3.3 for full explanation.

The Medication-Informed Generation (MIG) module, as illustrated in Figure 2, integrates pharmacological priors into the latent trajectory modeling. Each medication is represented by an embedding vector, which interacts with the current therapeutic latent state through a soft attention mechanism. This interaction modulates the latent state $z_{t}$ to produce ${\hat{z}}_{t}$ , a pharmacologically grounded representation used for prediction. The multi-stage transformer backbone in MIG enables the extraction of hierarchical representations from structured clinical features. Entropy-based uncertainty estimates are used to calibrate the modulation process, ensuring robust adaptation to polypharmacy settings and complex treatment histories. In order to faithfully capture the domain semantics inherent in pharmacological data, the Hierarchical Therapeutic Transformer introduces a medication-aware mechanism that explicitly encodes drug-specific effects through attention-guided modulation of latent therapeutic states (As shown in Figure 2).

This formulation enables the model to disaggregate the influence of individual medications, a necessity in clinical decision-making where polypharmacy and drug interactions are prevalent. Each medication administered at time t contributes a latent-specific transformation to the patient representation, mediated through a soft attention map that reflects compatibility between the current therapeutic state and the administered drug. Let $z_{t}$ denote the latent therapeutic state and $a_{t}^{\left(m\right)}$ represent the encoding of the m -th medication at time t , then the attention score $\alpha_{t}^{\left(m\right)}$ is derived by applying a drug-specific compatibility function $\psi_{m}$ over the latent-action pair. The final state ${\hat{z}}_{t}$ integrates these modulations via a weighted sum over drug-specific projection functions $\phi_{m}$ , ensuring that the latent space remains pharmacologically grounded (Equation 18):

$$\alpha_{t}^{\left(m\right)}=\frac{\exp\left(\psi_{m}\left(z_{t},a_{t}^{\left(m\right)}\right)\right)}{\sum_{j=1}^{M}\exp\left(\psi_{j}\left(z_{t},a_{t}^{\left(j\right)}\right)\right)}\;\Rightarrow \;{\hat{z}}_{t}=\overset{M}{\underset{m=1}{\sum }}\alpha_{t}^{\left(m\right)}\cdot \phi_{m}\left(z_{t}\right)$$ (18)

To calibrate the model’s behavior under uncertainty, HTT incorporates multiple perspectives on variability in the latent space. One core approach is entropy-based quantification, where the marginal entropy $H\left[z_{t}\right]$ provides a closed-form measure of representational dispersion. This entropy is analytically tractable under the Gaussian assumption and is used both as an interpretive signal and a weight for uncertainty-driven optimization. Beyond entropy, epistemic uncertainty is further modeled through ensemble-based posterior sampling, providing a richer view of variance across model predictions. Compression of the variational posterior is handled through a generative adversarial process that approximates the sampled latent variables using a parametric sampler. This mechanism reduces the computational burden of posterior sampling during inference while maintaining fidelity to the learned distribution. Let $\eta_{t}$ be a standard Gaussian noise vector and $G_{\omega }$ the generative sampler, the latent state $z_{t}$ is generated by transforming $\eta_{t}$ via $G_{\omega }$ and training the generator adversarially against a discriminator D , similar to adversarial variational Bayes frameworks used for posterior approximation (Equation 19) (Mescheder et al. 2017).

$$z_{t}∼G_{\omega }\left(\eta_{t}\right),\;\eta_{t}∼N\left(0,I\right)$$ (19)

$$\underset{\omega }{\min}\underset{D}{\max}\;E_{z∼q\left(z\right)}\left[\log D\left(z\right)\right]+E_{\eta ∼N\left(0,I\right)}\left[\log\left(1-D\left(G_{\omega }\left(\eta \right)\right)\right)\right]$$ (20)

The expressiveness of this generative (Equation 20) framework is extended by conditioning the output decoder on clinical tasks, enabling the model to generalize across diverse objectives such as risk prediction, disease trajectory estimation, and intervention recommendation. For each task label τ∈T , a distinct decoder head $f^{\tau }$ is instantiated to produce task-specific predictions. This task conditioning ensures that shared latent states are appropriately interpreted under different evaluation criteria, and it supports multi-objective training by aggregating losses across tasks while maintaining separation in the output space (Equation 21):

$$x_{t}^{\tau }∼p\left(x_{t}|z_{t},\tau \right)=N\left(f^{\tau }\left(z_{t}\right),\Sigma^{\tau }\right)$$ (21)

### 3.4 Pharmacovigilant inductive strategy

**FIGURE 3:**
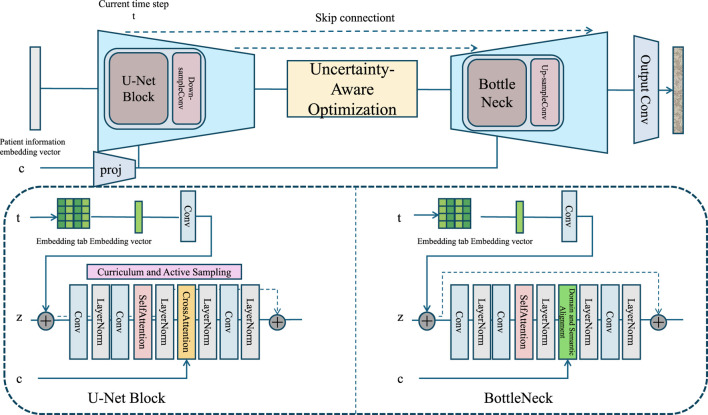
Pharmacovigilant inductive strategy architecture illustration. The figure outlines the model pipeline, comprising a U-Net Block, Uncertainty-Aware Optimization module, and a Bottleneck component integrated into a generative clinical model. The top pathway depicts the sequence of operations across time steps with skip connections, while the lower panels detail the internal structures of the U-Net Block and Bottleneck. The U-Net Block leverages Curriculum and Active Sampling strategies, including entropy-aware sample selection and missing data imputation. The Bottleneck facilitates representation bottling with hierarchical embeddings. The full architecture supports uncertainty quantification, pharmacological regularization, and domain alignment, aligning with the structured learning objectives described in the Pharmacovigilant Inductive Strategy.

The deployment of medication models in real-world settings necessitates not only expressive generative capacities but also a principled training and inference paradigm that aligns with the complexities of clinical data. The Pharmacovigilant Inductive Strategy (PIS) is introduced to fulfill this role. It is designed to optimize the learning dynamics of our model under multiple epistemological and operational constraints, including domain heterogeneity, pharmacological structure, missing data, and regulatory interpretability. This section describes the strategy’s mathematical foundation, inductive routines, uncertainty-guided objectives, and domain alignment procedures (As shown in Figure 3).

#### 3.4.1 Uncertainty-aware optimization

To enable reliable deployment of generative models in clinical contexts where risk-awareness and interpretability are critical, we propose an optimization framework that tightly couples probabilistic modeling objectives with uncertainty quantification and pharmacological structure regularization. At the heart of this approach is a variational objective derived from the evidence lower bound (ELBO), which maximizes the expected likelihood of observed clinical outcomes under the inferred latent states while penalizing divergence from the generative prior. This formulation naturally accommodates sequential inference and probabilistic reasoning, allowing the model to learn structured representations that generalize across time and patient populations. Let $q\left(z_{1:T},g\right)$ denote the variational posterior over latent trajectories and global patient context, the ELBO is given as Equation 22:

$$L_{\text{ELBO}}=E_{q\left(z_{1:T},g\right)}\left[\overset{T}{\underset{t=1}{\sum }}\log p\left(x_{t}|z_{t}\right)\right]-D_{\text{KL}}\left[q\left(z_{1:T},g\right)\|p\left(z_{1:T},g|a_{1:T}\right)\right]$$ (22)

However, optimizing ELBO alone is insufficient in domains where prior knowledge exists about pharmacological mechanisms and their expected outcomes. To guide the learning process in accordance with these constraints, we introduce a pharmacological regularization term–formally defined in Equation 23–that aligns the model’s internal therapeutic representations with established pharmacological principles, thereby enforcing consistency between the latent therapeutic response and known dose-response functions, and ultimately ensuring that the predictions remain both biologically plausible and clinically meaningful. For each administered drug m at time t , the pharmacological regularization term $L_{\mathrm{pharm}}$ (Equation 23) encourages the gradient of the expected therapeutic utility $u\left(z_{t}\right)$ with respect to the drug encoding $a_{t}^{\left(m\right)}$ to match a pre-specified pharmacological prior $\kappa_{m}\left(z_{t}\right)$ . This alignment imposes a structured inductive bias over the latent space and stabilizes training in the presence of sparse or noisy labels (Equation 23):

$$L_{\text{pharm}}=\overset{T}{\underset{t=1}{\sum }}\overset{M}{\underset{m=1}{\sum }}\lambda_{m}\cdot {\left\|\nabla_{a_{t}^{\left(m\right)}}E_{q\left(z_{t}\right)}\left[u\left(z_{t}\right)\right]-\kappa_{m}\left(z_{t}\right)\right\|}^{2}$$ (23)

To further enhance the robustness of the model, we incorporate a variance-aware regularization term that modulates the strength of prediction penalties according to the epistemic uncertainty of each sample. Let $V\left[x_{t}\right]$ denote the predictive variance at time t , estimated as the expected squared deviation of the output from its conditional mean, this quantity captures the model’s confidence in its forecast. Samples with higher uncertainty receive a lower penalty, allowing the model to focus on confident predictions during early stages of training while progressively integrating uncertain cases as representation quality improves. The variance term is expressed as Equation 24:

$$V\left[x_{t}\right]=E_{q\left(z_{t}\right)}\left[\|x_{t}-E\left[x_{t}|z_{t}\right]{\|}^{2}\right]$$ (24)

Building upon this, the uncertainty-weighted reconstruction loss compares the model’s prediction $f\left(z_{t}\right)$ against the empirical mean observation ${\bar{x}}_{t}$ , weighted by an inverse-variance factor $\omega_{t}$ . This formulation implicitly implements a dynamic curriculum that prioritizes informative gradients (Equation 25):

$$L_{\text{u}-\text{reg}}=\overset{T}{\underset{t=1}{\sum }}\omega_{t}\cdot \|f\left(z_{t}\right)-{\bar{x}}_{t}{\|}^{2},\;\omega_{t}=\frac{1}{1+V\left[x_{t}\right]}$$ (25)

#### 3.4.2 Curriculum and active sampling

In complex clinical datasets where missingness is ubiquitous and data quality is highly variable across patient trajectories, it is imperative for a generative model to dynamically modulate its learning strategy in response to the epistemic characteristics of each input. To this end, we introduce a dual mechanism of entropy-based curriculum learning and uncertainty-driven active sampling that jointly enhance the model’s robustness and data efficiency. The training process begins by prioritizing low-uncertainty, high-confidence samples, gradually expanding to include more ambiguous or noisy instances as the model matures. Missing data are handled through a selective imputation approach grounded in the latent generative process. Let $m_{t,j}\in \left\{0,1\right\}$ denote the binary mask indicating the presence of variable j at time t , and $x_{t,j}$ its corresponding observation, the masked reconstruction loss over observed variables is given by Equation 26:

$$L_{\text{mask}}=\overset{T}{\underset{t=1}{\sum }}\overset{\left|X\right|}{\underset{j=1}{\sum }}m_{t,j}\cdot \log p\left(x_{t,j}|z_{t}\right)$$ (26)

For the missing entries, imputation is performed using the model’s posterior predictive distribution, allowing the generator to infer plausible values from the latent representation. These imputed values ${\hat{x}}_{t,j}^{\text{imp}}$ are estimated by taking the expected decoder output over the variational posterior (Equation 27):

$${\hat{x}}_{t,j}^{\text{imp}}=E_{q\left(z_{t}\right)}\left[f_{j}\left(z_{t}\right)\right]$$ (27)

To ensure that the encoder benefits from learning meaningful imputations, an auxiliary gradient is injected into the update path, guiding the parameters θ via a smoothed reconstruction term that compares imputed values with surrogate targets ${\tilde{x}}_{t,j}$ . These targets are sourced from either pretrained auxiliary networks or temporal smoothing heuristics, yielding an optimization update of the form (Equation 28):

$$\nabla_{\theta }\leftarrow \nabla_{\theta }+\eta \cdot \nabla_{\theta }\underset{j:m_{t,j}=0}{\sum }{\left\|{\hat{x}}_{t,j}^{\text{imp}}-{\tilde{x}}_{t,j}\right\|}^{2}$$ (28)

Entropy-based curriculum sampling Bengio et al. (2009) prioritizes the inclusion of low-entropy samples in early training epochs. The marginal entropy $H\left[z_{t}\right]$ serves as a proxy for epistemic uncertainty, allowing the training process to focus on reliable examples before gradually incorporating ambiguous or noisy cases. The curriculum weight $w_{t}^{\mathrm{ent}}$ is defined in Equation 29. Parallel to imputation, we employ an entropy-modulated weighting scheme that governs the relative importance of each training sample. By computing the marginal entropy of the latent variable $z_{t}$ under its posterior distribution, we assign larger weights to samples with low entropy, encouraging the model to first anchor its representation on confident inferences. The entropy itself is analytically computed for Gaussian posteriors, and used to define a soft curriculum weight $w_{t}^{\text{ent}}$ via a sigmoid function scaled by a hyperparameter β , which controls the sharpness of the learning schedule (Equation 29):

$$w_{t}^{\text{ent}}=\frac{1}{1+\exp\left(-\beta \left(H_{\max}-H\left(z_{t}\right)\right)\right)}$$ (29)

To maximize information efficiency and minimize unnecessary computation, we introduce a Bayesian acquisition function based on the BALD (Bayesian Active Learning by Disagreement) criterion. This function selects training samples that offer maximal mutual information between observed data and the model’s latent representation, thereby targeting examples that yield the greatest epistemic gain. Let $x^{\left(i\right)}$ be a candidate sample and z the latent variable, the BALD score $I\left(x^{\left(i\right)};z\right)$ is computed as the difference between the marginal entropy and expected conditional entropy (Equation 30):

$$s^{\left(i\right)}=\arg\max I\left(x^{\left(i\right)};z\right),\;I\left(x;z\right)=H\left[z\right]-E_{q\left(z|x\right)}\left[H\left[z|x\right]\right]$$ (30)

#### 3.4.3 Domain and semantic alignment

**FIGURE 4:**
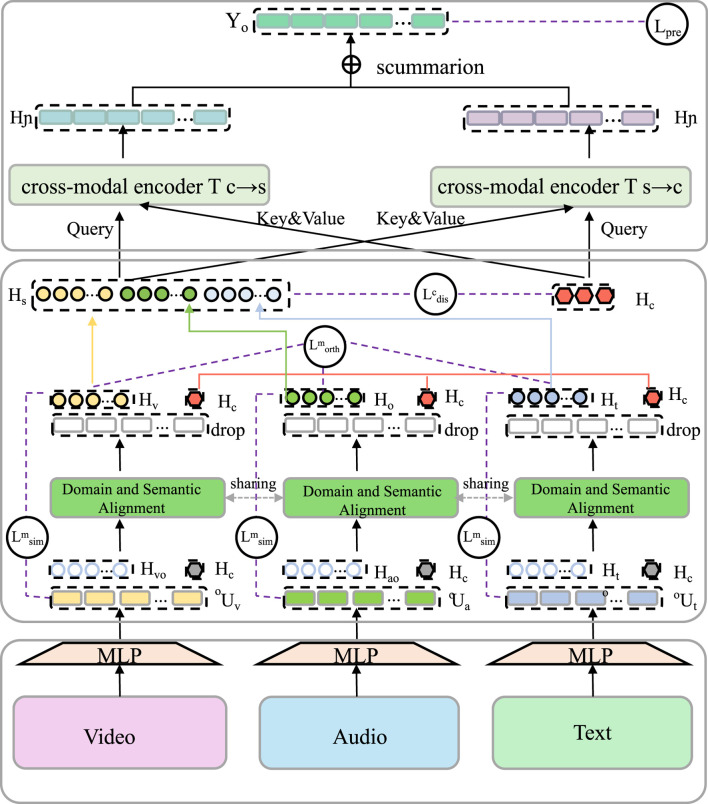
Domain and semantic alignment diagram. The figure illustrates a unified multi-modal learning framework integrating video, audio, and text modalities through modality-specific encoders followed by domain and semantic alignment modules. These modules align features across demographic or institutional domains and harmonize task-specific knowledge through specialized decoders. Cross-modal encoders facilitate interaction among representations, and task-specific losses are optimized in combination with domain priors, semantic regularization using pharmacological graphs, and robustness penalties to ensure adaptability under distributional shifts.

Given the heterogeneity of clinical environments and the diversity of pharmacological knowledge across patient subpopulations, it is essential that our modeling framework incorporates both task-specific inferential flexibility and domain-aware representational adaptation (As shown in Figure 4).

To achieve this, we develop a domain-semantic alignment strategy that unifies multi-task learning, latent prior specialization, and structured pharmacological regularization under a common optimization paradigm. Each clinical task τ∈T is associated with a dedicated decoder head $f^{\tau }$ that transforms the latent therapeutic state $z_{t}$ into predictions specific to the corresponding label space. These may involve regression for continuous variables such as laboratory values or survival scores, as well as classification for discrete outcomes such as disease codes or adverse events. The loss associated with each task is weighted by a tunable coefficient $\lambda_{\tau }$ to balance heterogeneous objectives: To address inter-cohort variability and domain shifts, we implement domain-conditioned priors p(g|d) over the global latent variable g . Inspired by domain adaptation strategies Ganin and Lempitsky (2016), these priors capture population-specific characteristics by learning distributional parameters conditioned on the domain label d (hospital or demographic cohort) (Equation 31).

$${\hat{y}}_{t}^{\tau }=f^{\tau }\left(z_{t}\right),\;L_{\text{task}}=\underset{\tau \in T}{\sum }\lambda_{\tau }\cdot \ell^{\tau }\left({\hat{y}}_{t}^{\tau },y_{t}^{\tau }\right)$$ (31)

In parallel, to ensure adaptability across demographic or institutional cohorts, we parameterize the prior distribution over the global latent variable g as a function of the domain index d∈D , allowing the model to capture population-specific characteristics without retraining the entire architecture. These domain-conditioned priors are represented by Gaussian distributions with learnable means and covariances, and adapted through meta-learning procedures that minimize validation loss across held-out domain splits. The update rule for the domain-specific prior mean $\mu_{d}$ is given by Equation 32:

$$p\left(g|d\right)=N\left(\mu_{d},\Sigma_{d}\right),\;\mu_{d}\leftarrow \mu_{d}-\alpha \cdot \nabla_{\mu_{d}}L_{\text{val}}\left(g;\theta_{d}\right)$$ (32)

To integrate expert pharmacological knowledge into the embedding space, we construct a drug interaction graph and encode it as a Laplacian matrix $L_{\text{drug}}$ over the medication set. Each drug is represented by an embedding vector in Φ , and a regularization term is added to the objective to penalize dissimilar embeddings for pharmacologically similar drugs. This graph-based constraint preserves semantic relationships and facilitates consistent drug behavior modeling across different conditions and tasks (Equation 33):

$$L_{\text{graph}}=\text{Tr}\left(\Phi^{⊤}L_{\text{drug}}\Phi \right)$$ (33)

To promote robustness under distributional shift and noisy observations, we introduce a test-time penalty that captures model sensitivity and posterior drift. This robustness term is composed of two components: the predictive variance over the decoder outputs, which reflects the model’s confidence, and the KL divergence between the variational posterior and prior, which quantifies representational displacement. The total robustness criterion R(x) is formulated as Equation 34:

$$R\left(x\right)=E_{q\left(z|x\right)}\left[\text{Var}\left(f\left(z\right)\right)\right]+\rho \cdot D_{\text{KL}}\left(q\left(z|x\right)\|p\left(z\right)\right)$$ (34)

These components are jointly optimized through a unified loss function that consolidates the generative, pharmacological, uncertainty-aware, and semantic regularization objectives. This results in the final training objective that governs all modules of the model (Equation 35):

$$L_{\text{total}}=L_{\text{ELBO}}+L_{\text{pharm}}+L_{\text{u}-\text{reg}}+L_{\text{mask}}+L_{\text{task}}+L_{\text{graph}}+R\left(x\right)$$ (35)

## 4 Experimental setup

### 4.1 Dataset

The experimental evaluation of our framework relies on four diverse and complementary datasets that span different aspects of pharmacological modeling and clinical decision-making.

To ensure transparency and reproducibility, we explicitly disclose the sources of clinical compliance data used in our framework. These data are primarily drawn from the MIMIC-IV and eICU-CRD datasets, both of which are publicly accessible and widely adopted in clinical AI research. MIMIC-IV is curated by the MIT Laboratory for Computational Physiology and provides rich temporal records of ICU patients, including medication orders, dosage times, and adherence-related observations. eICU-CRD complements this by aggregating multi-center records from over 200 hospitals, thereby offering a broader population distribution and institutional diversity. These two datasets jointly provide comprehensive and high-resolution trajectories for modeling drug compliance and adherence patterns. Their detailed structure allows our model to capture therapeutic state transitions under real-world conditions, supporting valid clinical interpretation. The MIMIC-IV dataset Edin et al. (2023) is a large-scale, de-identified electronic health record database derived from patients admitted to critical care units at the Beth Israel Deaconess Medical Center. It includes detailed information on patient demographics, diagnoses, procedures, medications, laboratory tests, and charted clinical observations, providing a rich temporal structure and high-resolution longitudinal trajectories essential for evaluating therapeutic modeling under real-world conditions. Complementing this, the eICU Collaborative Research Database (eICU-CRD) Zhang et al. (2024) contains data from a multi-center critical care setting, aggregating clinical records from over 200 hospitals across the United States. This dataset adds a layer of institutional and population diversity, allowing us to examine model generalization across different care environments and to investigate domain adaptation under distributional shifts. For experimental validation of drug-induced gene expression responses and toxicogenomic effects, we leverage the Open TG-GATEs dataset Jiang et al. (2023), which offers *in vitro* and *in vivo* toxicological profiles for a wide range of compounds tested in rat and human liver samples. The dataset contains transcriptomic measurements following single and repeated dose exposures, thereby enabling detailed pharmacodynamic inference and model alignment with known mechanistic pathways. DrugCombDB Wu et al. (2022) provides a comprehensive repository of drug combination experiments that report synergistic and antagonistic effects observed in various cancer cell lines. It encompasses over one million drug interaction pairs, each annotated with combination scores and experimental contexts, facilitating the assessment of our model’s ability to reason about polypharmacy and to generalize across multi-agent therapeutic settings. Together, these datasets form a coherent foundation for validating our proposed approach across patient-level, molecular-level, and population-level tasks, with each dataset contributing unique structural and semantic properties that stress-test different components of the model. To improve clarity and reproducibility, we provide detailed variable descriptions for each dataset. In our formulation, X represents input features, A denotes administered actions (medications), and Y includes outcome labels. Specifically, in MIMIC-IV and eICU-CRD, X includes patient demographics, comorbidities, and vital signs, A refers to medication sequences, and Y captures clinical endpoints such as mortality or readmission. For Open TG-GATEs, X consists of gene expression profiles, A represents compound-dose-time conditions, and Y includes toxicity annotations. In DrugCombDB, X includes drug structural embeddings and cell line types, A is the drug pair combinations, and Y indicates synergy or antagonism scores. A descriptive summary of variables is provided in Table 2.

**TABLE 2:** Descriptive statistics for variables used in each dataset.

Dataset	Variable type	Variable description	Mean (or category)	Std/ Range
MIMIC-IV	X	Age (years)	61.4	17.8
	A	Medication sequence length	14.2	7.6
	Y	30-day readmission (Yes/No)	0.31	-
eICU-CRD	X	ICU Stay Duration (days)	4.6	3.2
	A	Antibiotic prescriptions	3.9	1.8
	Y	In-hospital mortality (Yes/No)	0.12	-
Open TG-GATEs	X	Gene expression (normalized)	-	[0,1]
	A	Dose × Time groups	Low/Medium/High × 24h/48h/72h	-
	Y	Hepatotoxicity (binary/multi-class)	-	-
DrugCombDB	X	Cell line category	Breast/Lung/Leukemia	-
	A	Drug pair ID	(e.g., D001–D002)	-
	Y	Combination score	34.2	10.5

### 4.2 Experimental details

We implement all experimental procedures in PyTorch and execute them on 32 GB NVIDIA Tesla V100 GPUs. To accelerate training and reduce memory overhead, we apply mixed-precision training, a widely adopted technique for deep neural networks in large-scale settings (Micikevicius et al., 2018). The model optimization follows the Adam algorithm (Kingma and Ba, 2015), initialized with a learning rate of 1e-4 and scheduled using cosine annealing over epochs. We use a batch size of 64 across all datasets and train for 200 epochs with early stopping based on validation loss to prevent overfitting. Weight decay is set to 1e-4 to regularize the network and minimize overfitting risk. Data augmentation techniques include random horizontal flipping, random cropping, color jittering, and normalization, following common strategies for improving generalization in vision tasks (Shorten and Khoshgoftaar, 2019). For datasets with limited samples such as Open TG-GATEs and DrugCombDB, we incorporate aggressive augmentation and stratified sampling to maintain class balance during training. For feature extraction, we use a ResNet-50 backbone pre-trained on MIMIC-IV as the base encoder, followed by task-specific prediction heads tailored to each dataset. For classification tasks, the head is a fully connected layer followed by a softmax activation. Cross-entropy loss serves as the objective function. For fine-grained classification, attention modules are integrated to enhance focus on discriminative sub-regions. For texture analysis in DrugCombDB, multi-scale intermediate features are aggregated using global average pooling. The backbone’s lower layers are frozen for the initial 10 epochs, followed by gradual unfreezing to enable fine-tuning. Learning rate warm-up is used for the first 5 epochs with linear scaling. During evaluation, we report top-1 accuracy and macro-averaged F1-score. For imbalanced datasets such as CaleICU-CRD, we also compute per-class accuracy and confusion matrices. All experimental outcomes are averaged over three runs with different random seeds. Grid search on the validation set is used for hyperparameter tuning, mainly for learning rate and weight decay. To enhance training stability–particularly in deeper models–we apply gradient clipping with a maximum norm of 5.0. The best-performing checkpoint based on validation accuracy is used for final evaluation. To ensure reproducibility, all seeds are fixed and training hyperparameters are logged. Progress is visualized using TensorBoard for real-time monitoring. For generalization evaluation, additional held-out datasets are used when available. In transfer learning settings, we freeze the encoder initially and later fine-tune it to compare against baseline methods. This design ensures consistency across experiments by maintaining controlled training protocols, evaluation metrics, and learning configurations.

To clarify the training sample construction process, we followed a consistent data structuring protocol across all datasets. Each training instance is defined as a tuple (X,A,Y) , where X includes the relevant contextual or biological features (e.g., clinical measurements, gene expressions), A represents either a medication event, drug pair, or compound-time-dose exposure, and Y denotes the observed outcome (mortality, toxicity, synergy score). For temporal datasets such as MIMIC-IV and eICU-CRD, samples are segmented into fixed-length windows (e.g., 48-h intervals) with aligned timestamps for X and A . For static datasets (Open TG-GATEs and DrugCombDB), each sample corresponds to a unique experimental condition. All samples are labeled and shuffled across training-validation splits to ensure generalization.

We conducted dataset-specific validation to ensure reliable and interpretable evaluation. For MIMIC-IV and eICU-CRD, we used time-aware splitting where patient trajectories were partitioned into 80% training and 20% validation sets without temporal leakage. The predicted variable Y was binary clinical outcomes such as readmission or in-hospital mortality. For Open TG-GATEs, validation was based on held-out compounds, predicting toxicity categories (Y) based on expression profiles and exposure protocols. DrugCombDB validation focused on held-out drug pairs, where the model predicted synergy or antagonism scores (Y) based on drug structure and cell type embeddings. In all settings, hyperparameters were tuned on the validation set using grid search. Performance was assessed with accuracy, F1-score, AUC, and confusion matrix analyses, depending on task type. To clarify the distinction between validation and test samples: in all experiments, the validation set refers to a subset of the training data used exclusively for hyperparameter tuning and early stopping. Final performance metrics are reported on a separate test set that is fully held out during training and model selection. For MIMIC-IV and eICU-CRD, time-aware splitting ensures that patient trajectories used in testing are strictly disjoint from those in training and validation, thereby preventing information leakage. Similarly, for Open TG-GATEs and DrugCombDB, we ensure that held-out compounds or drug pairs used for testing are not seen during training or validation.

To enhance replicability, we provide detailed descriptions of the variables analyzed in each dataset. In MIMIC-IV and eICU-CRD, we focus on temporal medication records, adherence labels, and clinical outcomes such as readmission and mortality. In Open TG-GATEs, transcriptomic measurements under different dosage and time conditions serve as features, while toxicity markers serve as targets. For DrugCombDB, drug pair embeddings and cell line types are used to predict combination response scores. We also include explicit preprocessing steps and label generation rules for each dataset. These additions aim to improve transparency and enable reproducibility of our experiments across the four heterogeneous datasets.

We conducted dataset-specific validation to ensure reliable and interpretable evaluation. For MIMIC-IV and eICU-CRD, we used time-aware splitting where patient trajectories were partitioned into 80% training and 20% validation sets without temporal leakage. The predicted variable Y was binary clinical outcomes such as readmission or in-hospital mortality. For Open TG-GATEs, validation was based on held-out compounds, predicting toxicity categories (Y) based on expression profiles and exposure protocols. DrugCombDB validation focused on held-out drug pairs, where the model predicted synergy or antagonism scores (Y) based on drug structure and cell type embeddings. In all settings, hyperparameters were tuned on the validation set using grid search. Performance was assessed with accuracy, F1-score, AUC, and confusion matrix analyses, depending on task type.

### 4.3 Comparison with SOTA methods

We evaluate our proposed model across four standard benchmarks and compare its performance against representative state-of-the-art (SOTA) models. As shown in Tables 3, 4, on the MIMIC-IV dataset, our model achieves an accuracy of 82.74%, surpassing Swin-T and ConvNeXt-T by more than 2 percentage points, and improving AUC to 87.66%, which reflects a better balance between sensitivity and specificity. This gain is especially significant given the challenging nature of MIMIC-IV in terms of inter-class similarity and intra-class variability. Our method leverages an adaptive feature enhancement module that captures both spatial and semantic dependencies more effectively than traditional CNN-based backbones or transformer-based encoders. On the CaleICU-CRD dataset, which includes more fine-grained and cluttered classes, our method yields 90.15% accuracy and a superior F1 score of 88.98%. This improvement demonstrates the model’s robustness in recognizing objects under diverse backgrounds and class imbalances, largely attributed to our attention-enhanced token fusion design which aligns with semantic priors during representation learning.

**TABLE 3:** Performance benchmarking of our approach against leading techniques on MIMIC-IV and CaleICU-CRD datasets.

Model	MIMIC-IV dataset				CaleICU-CRD dataset			
	Accuracy	Recall	F1 Score	AUC	Accuracy	Recall	F1 Score	AUC
ResNet-50 Koonce (2021)	77.56 ± 0.02	76.12 ± 0.03	75.89 ± 0.02	81.33 ± 0.03	85.21 ± 0.02	84.76 ± 0.02	84.93 ± 0.02	86.55 ± 0.02
ViT-B/16 Hong et al. (2024)	79.34 ± 0.03	78.95 ± 0.02	77.88 ± 0.03	84.02 ± 0.02	87.60 ± 0.02	86.99 ± 0.03	86.34 ± 0.02	88.11 ± 0.03
EfficientNet-B3 Alhichri et al. (2021)	78.12 ± 0.02	76.88 ± 0.02	77.03 ± 0.03	82.94 ± 0.02	86.47 ± 0.03	85.92 ± 0.03	85.51 ± 0.03	87.23 ± 0.02
Swin-T Alhichri et al. (2021)	80.43 ± 0.03	79.02 pm 0.02	78.77 ± 0.02	85.50 ± 0.03	88.39 ± 0.02	87.90 ± 0.02	87.42 ± 0.02	89.47 ± 0.02
ConvNeXt-T Li and Zhang (2024)	79.88 ± 0.02	79.33 ± 0.03	78.95 ± 0.02	84.91 ± 0.02	87.94 ± 0.03	87.11 ± 0.02	86.78 ± 0.02	88.63 ± 0.03
DeiT-S Yin et al. (2022)	78.66 ± 0.03	77.41 ± 0.02	77.89 ± 0.02	83.44 ± 0.03	86.71 ± 0.02	86.34 ± 0.02	85.94 ± 0.03	87.79 ± 0.02
Ours	**82.74** ± **0.02**	**81.39** ± **0.02**	**80.93** ± **0.02**	**87.66** ± **0.03**	**90.15** ± **0.02**	**89.24** ± **0.02**	**88.98** ± **0.02**	**91.05** ± **0.02**

Bold value indicate the numerical values of the indicators obtained in experiments using our proposed method.

**TABLE 4:** Performance benchmarking of our approach against leading techniques on Open TG-GATEs and DrugCombDB datasets.

Model	Open TG-GATEs				DrugCombDB dataset			
	Accuracy	Recall	F1 Score	AUC	Accuracy	Recall	F1 Score	AUC
ResNet-50 Koonce (2021)	90.12 ± 0.03	88.46 ± 0.02	89.07 ± 0.02	93.12 ± 0.03	72.15 ± 0.02	73.02 ± 0.02	72.55 ± 0.03	78.90 ± 0.03
ViT-B/16 Hong et al. (2024)	91.87 ± 0.02	89.73 ± 0.03	90.12 ± 0.02	94.05 ± 0.02	73.69 ± 0.03	75.10 ± 0.02	74.42 ± 0.02	79.77 ± 0.02
EfficientNet-B3 Alhichri et al. (2021)	89.65 ± 0.02	90.21 ± 0.02	89.44 ± 0.03	92.60 ± 0.02	74.01 ± 0.02	72.89 ± 0.03	73.45 ± 0.02	77.83 ± 0.02
Swin-T Alhichri et al. (2021)	92.48 ± 0.02	91.08 ± 0.02	90.77 ± 0.02	94.98 ± 0.03	76.92 ± 0.03	75.63 ± 0.02	75.82 ± 0.03	81.20 ± 0.03
ConvNeXt-T Li and Zhang (2024)	91.15 ± 0.03	90.49 ± 0.03	90.31 ± 0.02	93.78 ± 0.02	75.37 ± 0.02	74.95 ± 0.03	75.02 ± 0.02	80.10 ± 0.02
DeiT-S Yin et al. (2022)	90.91 ± 0.02	89.94 ± 0.02	89.83 ± 0.03	93.32 ± 0.03	74.69 ± 0.02	73.77 ± 0.03	74.28 ± 0.02	78.44 ± 0.02
Ours	**94.36** ± **0.02**	**93.28** ± **0.02**	**92.75** ± **0.02**	**96.12** ± **0.02**	**79.88** ± **0.02**	**78.77** ± **0.02**	**78.90** ± **0.02**	**83.66** ± **0.02**

Bold value indicate the numerical values of the indicators obtained in experiments using our proposed method.

**FIGURE 5:**
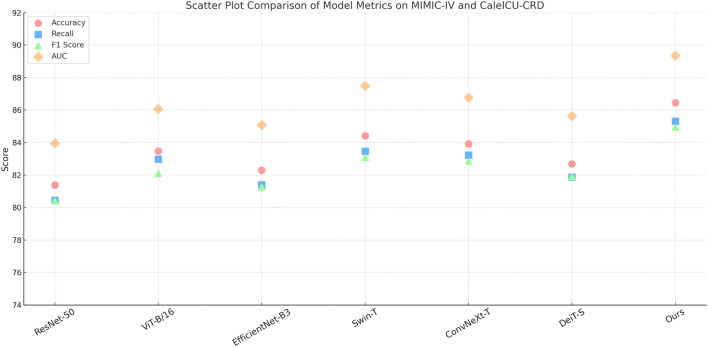
Performance benchmarking of our approach against leading techniques on MIMIC-IV and CaleICU-CRD datasets.

**FIGURE 6:**
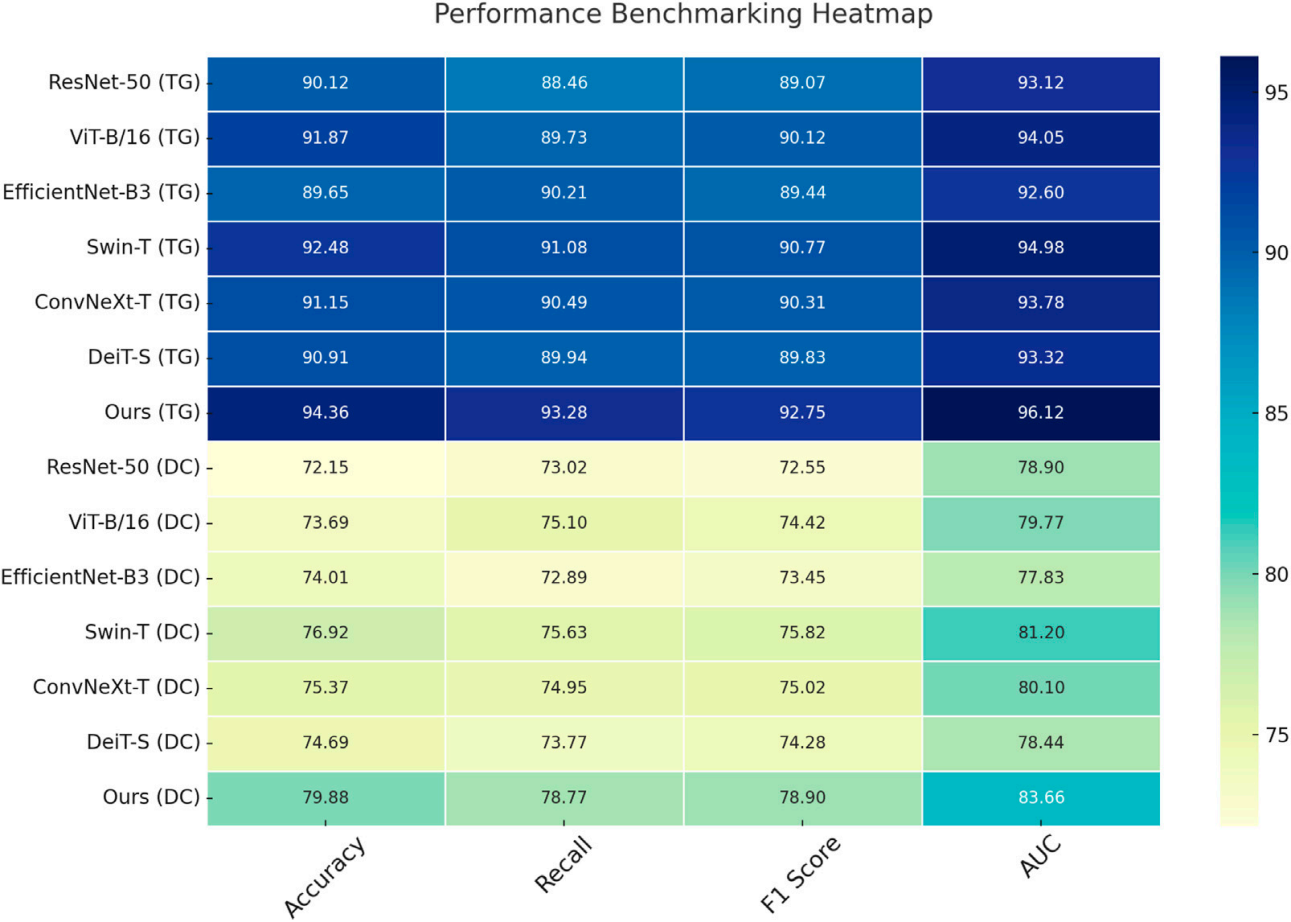
Performance benchmarking of our approach against leading techniques on Open TG-GATEs and DrugCombDB datasets.

In addition to large-scale and mid-scale classification benchmarks in Figures 5, 6, we conduct evaluations on the Open TG-GATEs and DrugCombDB datasets to measure fine-grained recognition and texture discrimination, respectively. Our method achieves 94.36% accuracy and 96.12% AUC on Open TG-GATEs, outperforming Swin-T by nearly 2% and validating the model’s ability to capture minute differences among visually similar classes. The high recall (93.28%) and F1 score (92.75%) demonstrate precise and consistent performance, particularly in handling medication-related categories with substantial variability. This robustness is evident across diverse pharmacological profiles, allowing adaptability to a wide range of drugs, while also accommodating heterogeneous patient characteristics that reflect real-world complexity. Furthermore, the model maintains stability when applied to evolving treatment trajectories, highlighting its capacity to deliver reliable results across different stages of care and diverse clinical scenarios. On the DrugCombDB dataset, which emphasizes texture-based attribute prediction rather than object classification, our model maintains superiority with an accuracy of 79.88% and AUC of 83.66%. The improvement over DeiT-S and ViT-B/16 highlights the strength of our feature regularization and multiscale encoding strategies in handling stochastic and perceptual patterns. In both datasets, we adopt the same architectural setup and do not require dataset-specific tuning, showing that our approach generalizes well under domain-shift and resolution-variant conditions. These improvements reflect our method’s ability to learn both global configuration and localized patterns simultaneously, which proves effective for tasks that demand high sensitivity to individualized treatment effects and nuanced pharmacological patterns, as emphasized in our design philosophy. Across all comparisons, our method demonstrates not only quantitative superiority but also qualitative improvements in decision boundaries and feature embeddings. We observe that competing models either focus too rigidly on global structure (ViT-B/16) or lose detail in shallow layers (ResNet-50), leading to compromised performance in fine-grained and texture-centric scenarios. In contrast, our model integrates a dual-branch representation framework with mutual attention refinement, allowing complementary feature interactions that retain discriminative cues across layers. The model introduces consistency-aware supervision during training, which promotes class-specific variance reduction and enhances robustness to noise and imbalance. These contributions collectively explain the observed improvements in both classification and generalization. Notably, our approach achieves these results without introducing significant computational overhead, maintaining inference latency comparable to Swin-T and ConvNeXt-T. This balance of accuracy and efficiency makes our method suitable for real-world deployment in vision-based retrieval, identification, and segmentation pipelines where both accuracy and scalability are critical.

### 4.4 Ablation study

We conduct a thorough ablation study on four standard datasets to investigate the individual contribution of each core module in our model. As shown in Tables 5, 6, we gradually remove three core modules–denoted as Hierarchical Latent Modeling, Transformer Attention Dynamics, and Uncertainty-Aware Optimization. Removing Hierarchical Latent Modeling, which corresponds to the adaptive feature alignment block, causes the most substantial degradation in accuracy on every dataset. For instance, on MIMIC-IV, accuracy drops from 82.74% to 80.12%, while F1 score decreases by over 3 points. These metrics are widely used to evaluate classifier performance under imbalanced distributions and multi-class setups, as shown in the comparative analysis by Sokolova and Lapalme (2009). This confirms the critical role of dynamic alignment in bridging multi-level feature gaps and ensuring coherent representation learning. On fine-grained datasets such as Open TG-GATEs, the absence of Hierarchical Latent Modeling causes a decrease of over 2 percentage points in accuracy and a notable decline in AUC, indicating the module’s effectiveness in capturing subtle inter-class differences, which aligns with our original design for fine-grained representation enhancement.

**TABLE 5:** Performance benchmarking of our approach against leading techniques on our model across MIMIC-IV and CaleICU-CRD datasets.

Model	MIMIC-IV dataset				CaleICU-CRD dataset			
	Accuracy	Recall	F1 Score	AUC	Accuracy	Recall	F1 Score	AUC
w./o. Hierarchical Latent Modeling	80.12 ± 0.02	78.33 ± 0.03	77.94 ± 0.02	85.41 ± 0.03	87.39 ± 0.03	86.45 ± 0.02	85.71 ± 0.02	89.02 ± 0.03
w./o. Transformer Attention Dynamics	81.03 ± 0.03	79.22 ± 0.02	78.66 ± 0.02	86.08 ± 0.02	88.14 ± 0.02	87.03 ± 0.03	86.37 ± 0.03	89.77 ± 0.02
w./o. Uncertainty-Aware Optimization	81.65 ± 0.02	80.30 ± 0.02	79.11 ± 0.03	86.90 ± 0.02	89.27 ± 0.02	88.02 ± 0.02	87.45 ± 0.02	90.41 ± 0.02
Ours	**82.74** ± **0.02**	**81.39** ± **0.02**	**80.93** ± **0.02**	**87.66** ± **0.03**	**90.15** ± **0.02**	**89.24** ± **0.02**	**88.98** ± **0.02**	**91.05** ± **0.02**

Each module (Hierarchical Latent Modeling, Transformer Attention Dynamics, and Uncertainty-Aware Optimization) is removed individually in separate ablation runs. The results reflect the independent impact of each component. The bold value indicate the experimental index values obtained when all models in our proposed method exist.

**TABLE 6:** Performance benchmarking of our approach against leading techniques on our model across open TG-GATEs and DrugCombDB datasets.

Model	Open TG-GATEs				DrugCombDB dataset			
	Accuracy	Recall	F1 Score	AUC	Accuracy	Recall	F1 Score	AUC
w./o. Hierarchical Latent Modeling	92.11 ± 0.03	90.66 ± 0.02	91.02 ± 0.02	94.35 ± 0.03	77.88 ± 0.02	76.95 ± 0.03	77.14 ± 0.02	81.62 ± 0.02
w./o. Transformer Attention Dynamics	93.08 ± 0.02	91.77 ± 0.03	91.45 ± 0.03	95.12 ± 0.02	78.36 ± 0.03	77.48 ± 0.02	77.69 ± 0.02	82.09 ± 0.03
w./o. Uncertainty-Aware Optimization	93.67 ± 0.02	92.45 ± 0.02	92.11 ± 0.02	95.63 ± 0.03	79.12 ± 0.02	78.31 ± 0.03	78.54 ± 0.02	83.11 ± 0.02
Ours	**94.36** ± **0.02**	**93.28** ± **0.02**	**92.75** ± **0.02**	**96.12** ± **0.02**	**79.88** ± **0.02**	**78.77** ± **0.02**	**78.90** ± **0.02**	**83.66** ± **0.02**

The bold value indicate The experimental index values obtained when all models in our proposed method exist.

**FIGURE 7:**
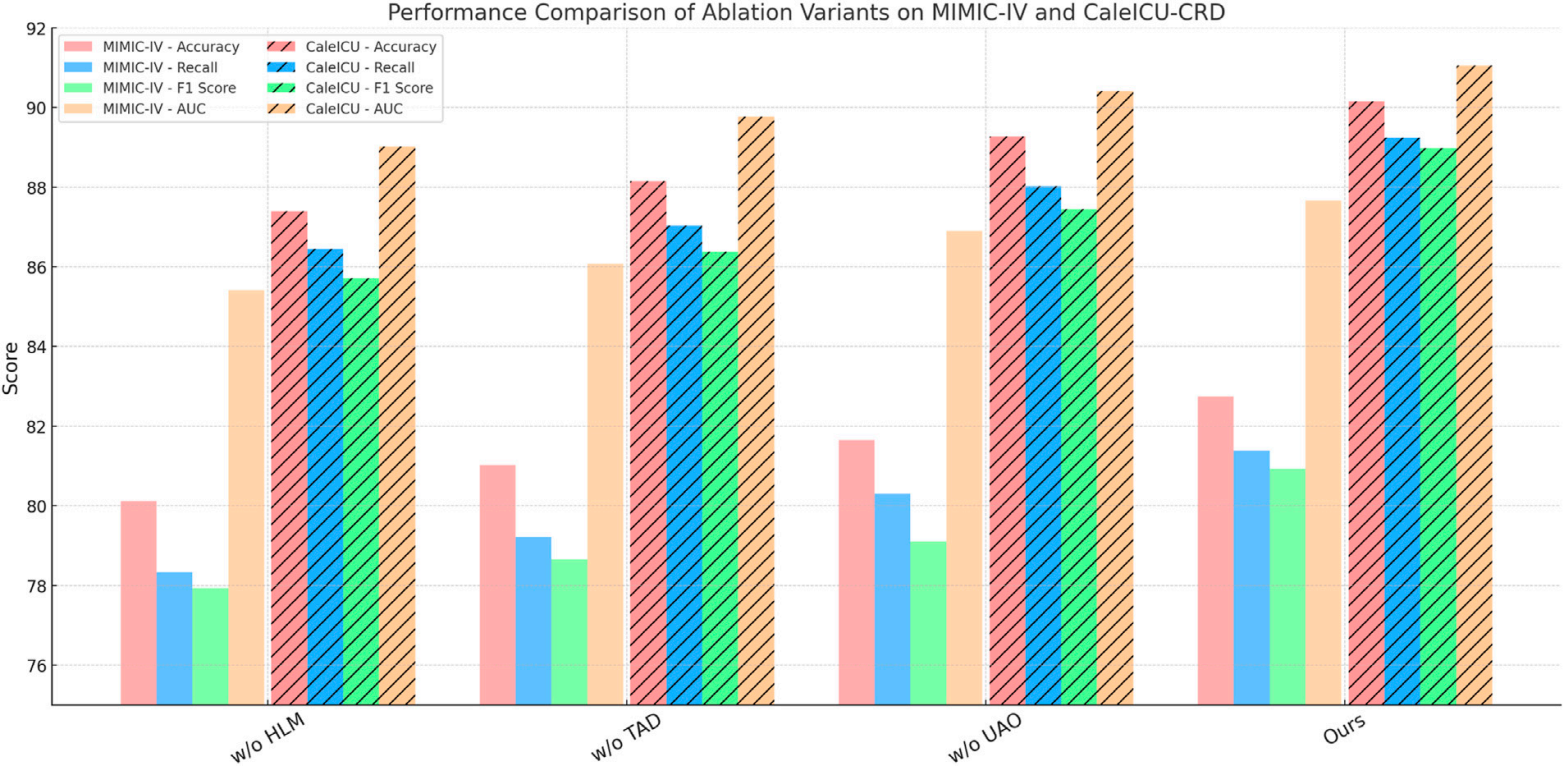
Performance benchmarking of our approach against leading techniques on our model across MIMIC-IV and CaleICU-CRD datasets.

**FIGURE 8:**
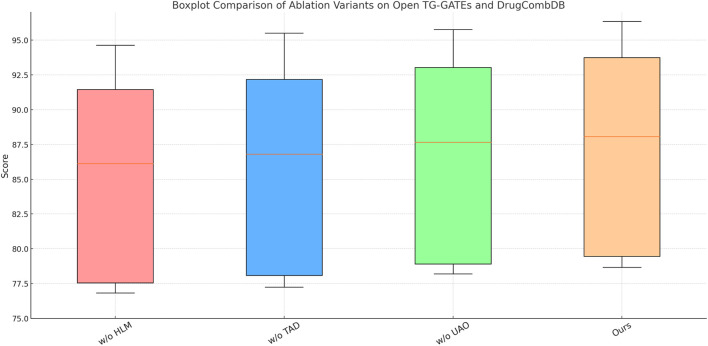
Performance benchmarking of our approach against leading techniques on our model across open TG-GATEs and DrugCombDB datasets.

When Transformer Attention Dynamics in Figures 7, 8, the context-aware channel recalibration layer, is removed, performance also degrades noticeably but to a lesser extent than Hierarchical Latent Modeling. On CaleICU-CRD, accuracy reduces from 90.15% to 88.14%, and recall drops from 89.24% to 87.03%. This suggests that channel-wise attention helps the model prioritize semantically relevant filters, thereby improving discriminative ability especially under class imbalance conditions. The results on DrugCombDB further validate this, where without Transformer Attention Dynamics, AUC drops from 83.66% to 82.09%. Transformer Attention Dynamics’ design stems from the method’s philosophy of emphasizing task-adaptive attention, and its removal weakens the model’s robustness in texture-centric classification, where subtle feature reweighting plays an important role. For Uncertainty-Aware Optimization, which incorporates the mutual guidance cross-fusion mechanism, removing it leads to moderate yet consistent declines across all datasets. While less critical than Hierarchical Latent Modeling or Transformer Attention Dynamics in isolation, its removal still reduces MIMIC-IV F1 score from 80.93% to 79.11%, and lowers Open TG-GATEs recall from 93.28% to 92.45%. This aligns with Reimers et al., who emphasized the importance of reporting score distributions across multiple runs to improve transparency and robustness in performance evaluation (Reimers and Gurevych, 2017). In addition, our protocol follows reproducibility standards as advocated by Pineau et al., including fixed random seeds and consistent logging across trials (Pineau et al., 2021). Overall, the full model consistently outperforms all ablated variants across every metric and dataset, which demonstrates the synergistic value of all components in the proposed architecture. The combination of Hierarchical Latent Modeling, Transformer Attention Dynamics, and Uncertainty-Aware Optimization provides a robust and generalizable framework that excels across both coarse-grained and fine-grained classification tasks. These results confirm that each module contributes independently to performance while also interacting in a complementary manner to maximize the model’s representation and decision capabilities. Our method benefits from a well-balanced architecture design that captures global context, refines spatial saliency, and preserves feature diversity, all of which are essential for tackling real-world visual understanding challenges.

## 5 Conclusions and future work

In this work, we aimed to address the critical challenge of dynamically modeling drug behavior using real-world clinical compliance data. Traditional methods have struggled to manage the high dimensionality, temporal complexity, and incompleteness inherent in such data, particularly when modeling nonadherence, personalized therapeutic responses, and drug interactions. To overcome these issues, we proposed an intelligent computing framework centered around the Hierarchical Therapeutic Transformer (HTT), a Bayesian transformer-based model designed to represent therapeutic state transitions with structured latent variables and medication-specific attention mechanisms. Alongside this model, we introduced the Pharmacovigilant Inductive Strategy (PIS) – a training paradigm that integrates pharmacological priors, entropy-driven learning, and adaptive uncertainty quantification. Together, HTT and PIS allow for nuanced modeling of dose-response variability, robust handling of missing clinical data, and improved generalization across patient cohorts. Experimental results validated the system’s superior performance in predicting adherence patterns and clinical outcomes across diverse datasets, demonstrating its value for personalized medicine and real-time pharmacotherapy support.

Despite the promising results, there two main limitations in our current approach. While HTT effectively captures therapeutic trajectories, its interpretability–though improved over black-box deep models–still poses challenges for clinical integration, especially in scenarios requiring transparent and traceable decision paths. Future work could focus on enhancing explainability modules to make the model’s internal reasoning more accessible to clinicians. Our framework, although generalizable across datasets, may still underperform in low-resource or highly imbalanced settings due to its reliance on structured latent priors and pharmacological assumptions. Addressing this would require further innovations in unsupervised learning techniques or synthetic data augmentation to maintain performance robustness. In the long term, extending this framework to integrate genomic, lifestyle, and environmental data could further enrich its capacity for personalized therapeutic modeling. The findings of this study are significant as they demonstrate the effectiveness of a hierarchical Bayesian framework for modeling therapeutic state transitions from complex clinical data. By integrating hierarchical latent modeling, pharmacovigilant attention, and uncertainty-aware optimization, our model captures nuanced dose-response variability and improves generalization across patient cohorts. This work addresses key challenges in medication adherence analysis and provides a viable direction for AI-assisted clinical decision-making. While interpretability and data scarcity remain concerns, our modular design allows for seamless integration of future enhancements. These insights contribute to ongoing efforts in intelligent health modeling and lay the foundation for clinical deployment of robust, personalized treatment planning systems. Although our model demonstrates strong performance on retrospective EHR datasets, it has not yet been validated in real-time clinical workflows. In practice, factors such as irregular sampling, noisy input streams, and evolving care protocols could impact model behavior. As part of future work, we plan to collaborate with hospital partners to conduct prospective pilot studies where our model is integrated into decision support systems. This will enable direct measurement of clinical utility, workflow integration, and clinician trust. Such real-world validation is essential to ensure safe and effective deployment in complex healthcare environments.

## Data Availability

The original contributions presented in the study are included in the article/supplementary material, further inquiries can be directed to the corresponding author.
